# Associated Pyoderma Gangrenosum, Erythema Elevatum Diutinum, and Chronic Recurrent Annular Dermatosis: The Neutrophilic Disease Spectrum

**DOI:** 10.7759/cureus.21005

**Published:** 2022-01-07

**Authors:** Maha Salih Alj, Madiha Eljazouly, Fatimazahra Chahboun, Abderahmane Al Bouzidi, Soumiya Chiheb

**Affiliations:** 1 Dermatology Unit, Cheikh Khalifa International University Hospital, Mohammed VI University of Health Sciences, Casablanca, MAR; 2 Pathology Unit, Cheikh Khalifa International University Hospital, Mohammed VI University of Health Sciences, Casablanca, MAR

**Keywords:** sweet’s syndrome, erythema elevatum diutinum, chronic recurrent annular dermatosis, pyoderma gangrenosum, neutrophilic dermatosis

## Abstract

Neutrophilic dermatoses (ND) refer to a group of skin diseases characterized histologically by a cutaneous infiltrate of mature polymorphonuclear cells without an identifiable cause. Previously described as autonomous, these clinically distinct entities are included in the spectrum of neutrophilic disease due to the existence of overlapping forms, as described in our observation. Erythema elevatum diutinum (EED) is a rare dermatosis characterized by reddish-violaceous to browning papulonodular and plaques and belongs to the spectrum of cutaneous leukocytoclastic vasculitis. Chronic recurrent annular neutrophilic dermatosis (CRAND) is an exceptional neutrophilic dermatosis characterized by chronic annular lesions and the absence of generalized signs or hematological abnormalities. Histological features are similar to those seen in Sweet’s syndrome.

A 55-year-old woman with a history of pyoderma gangrenosum (PG) presented successively with two rare forms of ND, namely, EED and CRAND. There were no clinical or paraclinical arguments for any underlying systemic disease. Treatment with azathioprine 100 mg/day and topical steroids led to a total regression of lesions after a nine-month follow-up.

Our observation is important because it reports two rare entities, CRAND and EED. Their occurrence in a single patient with a history of PG illustrates the concept of “neutrophilic disease” reported in the 1990s.

## Introduction

Neutrophilic dermatoses (ND) are a set of clinically heterogeneous diseases characterized by the presence of a histologic neutrophilic infiltration with no identifiable cause. Previously described as independent entities, they are now considered a continuous spectrum of clinical entities. This concept is supported by the existence of different forms of overlap, as evidenced by the case we report. Our patient presented successively with three forms of ND.

## Case presentation

**Figure 1:**
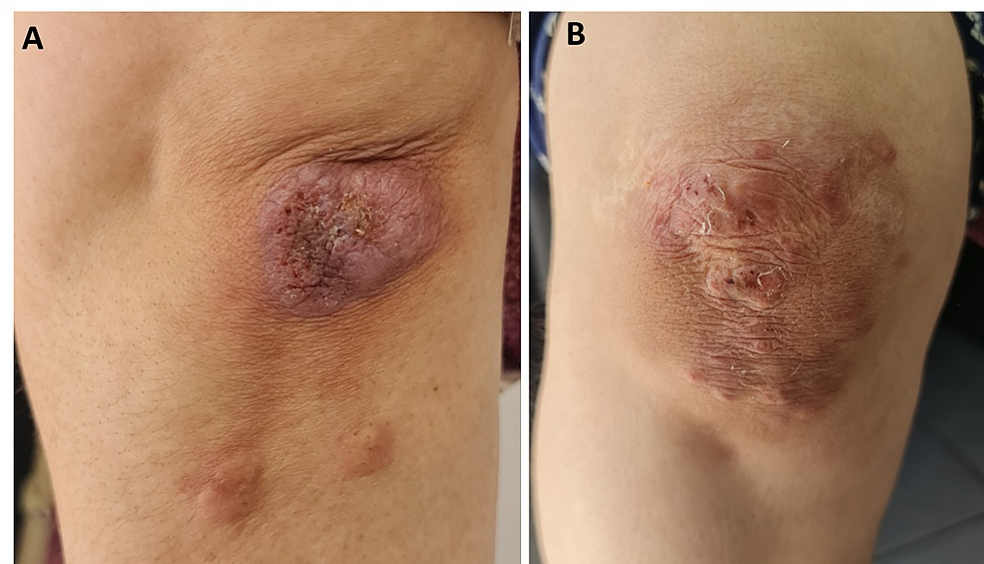
The erythematous and violaceous nodules and plaques on the patient’s elbows (A) and knees (B).

A 55-year-old woman was under follow-up for two years due to pyoderma gangrenosum (PG). The ulcers started as papulopustular lesions progressing to large painful ulcers localized on the breasts, knees, and buttocks, with no associated symptomatology. Oral corticosteroids were administered with a good initial response. However, the lesions recurred once the steroid dose was decreased. Subsequently, several drugs were administered, including colchicine, methotrexate, disulone, and thalidomide, with bad tolerance leading to their discontinuation. The evolution was marked by several episodes of relapses and remissions of her PG as well as the development of multiple erythematous and violaceous nodules and plaques on her elbows and knees (Figure 1).

**Figure 2:**
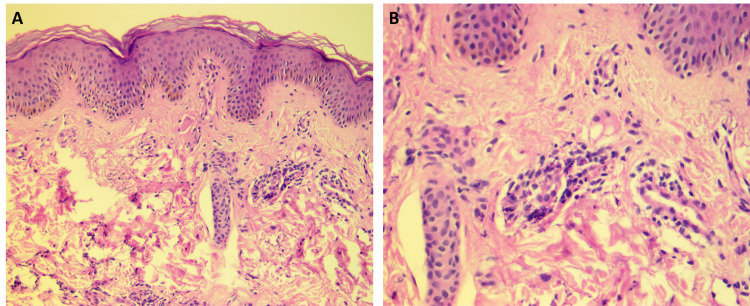
Erythema elevatum diutinum: histopathology from a skin biopsy on the elbow showing dermal leukocytoclastic vasculitis and diffuse neutrophilic infiltrate in the dermis (hematoxylin and eosin 20× (A) and 40× (B)).

Histopathology from a skin biopsy revealed dermal leukocytoclastic vasculitis and diffuse neutrophilic infiltrate in the dermis (Figure 2).

Histopathology findings revealed the diagnosis of erythema elevatum diutinum (EED). Hence, we initiated oral treatment with colchicine 1 mg/day and topical clobetasol 0.05%. One year later, she came back for the same EED lesions, which showed no regression. She also reported the occurrence of annular plaques with a centrifugal extension on the posterior surface of the legs and ankles. At this time, the patient described three previous episodes with similar lesions in the same area and the median surface of the left wrist, spontaneously resolving within four weeks.

**Figure 3:**
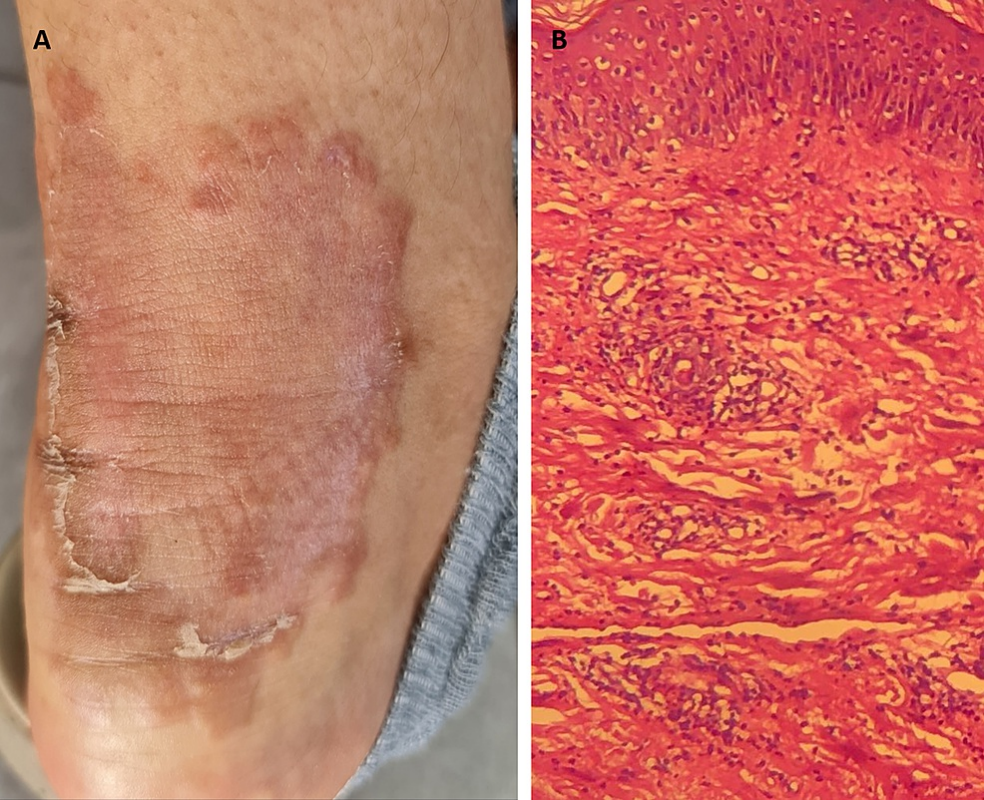
(A) Chronic erythematous lesion bordered by a papular ring with central healing associated with a fine scale. (B) Histopathology from a skin biopsy of the papular border showing papillary dermis edema associated with dense inflammatory infiltration rich in neutrophils in the superficial and medium dermis, without vasculitis (hematoxylin and eosin 40×).

The physical examination revealed an erythematous lesion on the posterior surface of the left leg and ankle bordered by a papular infiltrated ring with central healing associated with a fine scale (Figure 3).

**Figure 4:**
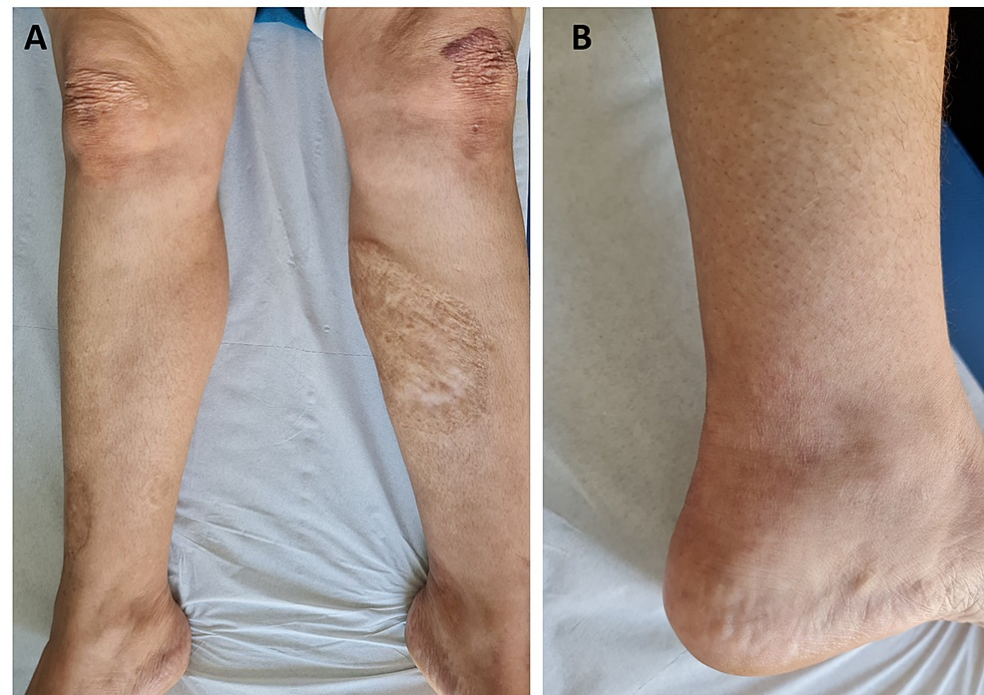
Complete regression of the lesions after a nine-month follow-up.

There were no transit disorders and no clinical or paraclinical arguments for underlying bowel inflammation, hematologic malignancy, or inflammatory rheumatism. Moreover, complete blood cell count, C-reactive protein, serum protein electrophoresis, rheumatoid factor, antinuclear antibodies, and the anti-cyclic citrullinated peptide level were normal. HIV and Borrelia serology results were negative. The upper and lower digestive endoscopic evaluation and histopathology of duodenal, terminal ileal, and colonic biopsies were normal. Histological skin examination from a biopsy of the papular border revealed papillary dermis edema associated with a dense inflammatory infiltration rich in neutrophils in the superficial and medium dermis, without vasculitis (Figure 3). The histological findings were compatible with the diagnosis of Sweet’s syndrome (SS). Based on the clinical and histological findings, the diagnosis of chronic recurrent annular dermatosis (CRAND) was made. Treatment with azathioprine 100 mg/day and topical treatment with clobetasol 0.05% was started. Lesions of PG, EED, and CRAND showed complete regression after a nine-month follow-up (Figure 4).

## Discussion

This report illustrates the concept of “neutrophilic disease” described by Wallach in 1990 and describes three neutrophilic pathologies occurring in the same patient, namely, PG, EED and CRAND.

CRAND was first described in 1989 by Christensen et al. [1]. This entity is usually described as a subtype of SS. To our knowledge, less than 10 cases have been reported. As described in our case, this condition occurs most frequently without any underlying diseases. However, other studies have reported cases of CRAND associated with a monoclonal gammapathy immunoglobulin (Ig)G k type, rheumatoid arthritis, and sarcoidosis [2]. This condition affects women after the age of 40. It is characterized by chronic, recurrent, and painless skin lesions. These lesions are annular with centrifugal progress within four weeks on average. Lesions may reappear within months to several years (one to five years). They are semiologically similar and occur near or at a distance from the initial lesion. The infiltrated papular border is centered by a fine scale. Histological features are similar to SS and show dense neutrophilic infiltrate and dermal edema [3].

The existence of overlapping types of ND has been described in several reports. The most frequent association concerns SS with PG without other underlying pathologies [4]. As described in our case, Caucanas et al. reported the development of three different entities of ND in one patient (EED, PG, and SS) [5]. PG can be associated with EED without other underlying pathologies [6], or with IgA monoclonal gammopathy [7] or myelodysplastic syndrome [8].

Although EED can occur at any age, it is most common in the fourth and sixth decades. As in our patient, the cutaneous lesions of EED typically affect the extensor surfaces of extremities and can be localized on the dorsal hands, elbows, knees, feet, and legs. They begin as erythematous nodular lesions and become more indurated, brown, and violaceus over time. Initially classified as ND, it is now considered a leukocytoclastic vasculitis according to its histopathological features. The pathogenesis of EED is poorly understood. It has been suggested that lesions appear secondary to the circulation of immune complexes that deposit continuously in blood vessels, leading to the activation of complement cascade and lecocytoclastic vasculitis. This pathophysiological mechanism is supported by a study of five patients with EED. Three patients presented with an increased C1q binding [9]. The pathologic findings are correlated with the age of the lesion. The recurrent nature and chronicity of EED represent the most effective way to differentiate it from other types of cutaneous vasculitis [10].

Several autoimmune and inflammatory diseases may be associated with EED, including bacterial and viral infections, inflammatory bowel diseases, systemic lupus erythematosus, rheumatoid arthritis, myelodysplastic syndrome, hairy cell leukemia, Waldenstrom’s macroglobulinemia, lymphoma, and solid malignancies [10]. Therefore, IgA gammopathy represents the most frequent disease associated with EED [11]. Yiannias et al. showed that EED lesions can precede elevated IgA levels and the development of myeloproliferative disorder by 7.8 years [12]. IgA can activate the alternate complement, and IgA aggregates have been found in the lesions [13]. Therefore, it is important to monitor EED patients with immunofixation electrophoresis. IgA anti-neutrophilic centromere antibody antibodies may be a neutrophilic activator seen in three independent reports [14-16].

Neutrophil-mediated skin diseases are considered autoinflammatory diseases since the discovering of numerous mutations involving different autoinflammatory genes. The activation of the innate immune system and its dysregulation results from overexpression of the proinflammatory cytokine interleukin (IL)-1 with the contributory role of IL-17 and other effector molecules [17]. These findings suggest new perspectives in the management of DN involving IL-1α blockade.

Although ND represent a continuum of different clinical entities, as evidenced by our observation, clinical responses to different drugs for their management are variable. Dapsone (100 mg/day) represents the first-line treatment of EED [13]. Treatment of CRAND is not codified due to the small number of reported cases. General short-term oral steroids at 30 mg/day or topical steroids may allow rapid regression of lesions without preventing their recurrence. Colchicine (1 mg/day) and dapsone (100 mg/day) individually or in combination with topical steroids are sometimes effective in the prevention of relapses [3]. PG is treated with systemic steroids as first-line treatment and cyclosporine (3-5 mg/kg/day) as second-line treatment. In our case, azathioprine (100 mg/day) in combination with topical steroids was effective in the management of the three reported entities. Therefore, azathioprine represents a good therapeutic alternative in this neutrophilic overlap scenario.

## Conclusions

The occurrence of PG, EED, and CRAND in the same patient supports the concept that these entities represent a spectrum of polygenic autoinflammatory conditions marked by overexpression of the proinflammatory cytokine IL-1. The frequent association with several underlying pathologies justifies complete evaluation to search for a systemic disease along with long-term monitoring.
